# Application of the pivot forward combined leveraging technique in the treatment of thoracolumbar fractures: a finite element analysis and clinical study

**DOI:** 10.3389/fsurg.2026.1941333

**Published:** 2026-09-15

**Authors:** Yan Zhou, Jianghua Ming, Ming Deng, Yonggang Ma

**Affiliations:** Department of Spine Surgery, Renmin Hospital of Wuhan University, Wuhan, China

**Keywords:** biomechanics, reduction technique, retrospective study, short segment fixation, thoracolumbar fracture

## Abstract

**Background:**

This study aimed to explore the three-dimensional finite element analysis and clinical effect of the pivot forward combined leveraging technique in the treatment of thoracolumbar fracture.

**Methods:**

Two internal fixation models were designed based on the pivot forward combined leveraging technique and traditional fixation technique for finite element analysis. Data of 47 patients who underwent pivot forward combined leveraging technique for thoracolumbar fracture were retrospectively analyzed. The vertebral body index (VBI), height of the anterior margin of injured vertebra (HAMIV), injured vertebra angle (IVA), kyphotic Cobb angle (KCA), the main outcome function included visual analog scale (VAS), and Oswestry disability index (ODI) before, after and at the last follow-up were measured.

**Results:**

The finite element results showed that the nail rod stress and nucleus pulposus stress measured in pivot forward combined leveraging technique group were significantly lower than those in the traditional fixation group. Clinically, the procedure of pivot forward combined leveraging technique was successfully completed in 47 cases. The patients' preoperative VBI, HAMIV, IVA, KCA, VAS and ODI scores were 0.64 ± 0.07, 0.60 ± 0.09, 23.70° ± 3.26°, 11.37° ± 9.53°, 8.04 ± 0.83, and 51.81 ± 9.21, respectively. Postoperative measurements were 0.93 ± 0.04, 0.96 ± 0.04, 4.22° ± 2.32°, −1.71° ± 8.07°, 3.26 ± 0.64 and 21.15 ± 5.52, respectively. At the last follow⁃up, the measurements were 0.91 ± 0.04, 0.94 ± 0.04, 5.49° ± 2.32°, 0.18° ± 8.25°, 1.00 ± 0.78, and 6.49 ± 1.90, respectively. Notably, a marked difference was obtained between the preoperative and postoperative data, as well as the preoperative and the last follow⁃up data (*P* < 0.05).

**Conclusions:**

The pivot forward combined leveraging technique has high biomechanical stability and can restore the height of the injured vertebra and correct the kyphosis angle of the injured vertebra.

## Introduction

Thoracolumbar fracture is one of the most common spinal injuries, caused by a fall or auto collision (1). Complications from a thoracolumbar fracture include paralysis, discomfort, deformity, and loss of function. The pedicle screw fixation technology can effectively treat thoracolumbar fractures and it is widely used (2, 3).

Although it is a tried-and-true method, pedicle screw fixation faces certain unresolved and limitations. The three columns are supported by the pedicle screw throughout the posterior fixation and reduction procedure, and the posterior longitudinal ligament's hinge action can reduce the fracture vertebra (4). However, the progression of kyphosis and the failure of internal post-operation are commonly reported (5, 6). The phenomenon is associated with the strong tension in the anterior longitudinal ligament which makes the posterior column undergo the most stretched when the three columns are stretched simultaneously. The posterior ligament complex is separated and stretched again, weaking the tension band effect on the basis of trauma, increasing the stress shield and the effect of metal internal fixator cantilever beam, inducing the aforementioned complications (7, 8).

In this study, we aimed to apply a finite element analysis and perform a clinical study to examine the potential of the pivot forward combined leveraging technique to treat thoracolumbar burst fractures. Here, we retrospectively analyzed the imaging data and evaluation indexes of thoracolumbar fracture patients with kyphotic angle >20° treated using the pivot forward combined leveraging technique in our hospital. The study aimed to (1) evaluate the biomechanical advantages and clinical efficacy of the pivot forward combined leveraging technique, (2) assess its effectiveness, safety, and indications in treating thoracolumbar fractures, and (3) analyze the key procedural aspects of this technique.

## Material and methods

### Intact finite element model

Mimics 19.0 modeling process: Geometrical details were derived from a 30-year-old healthy male volunteer in an unloaded neutral position through high-resolution computed tomography. The Dicom data which included five lumbar vertebrae and four intervertebral disks, was imported into the Mimics 19.0 sofware (Materialise Inc., Leuven, Belgium). First, grayscale values were adjusted to enhance contrast, followed by threshold-based segmentation. Redundant structural connection were eliminated using the “Edit Masks” command. Finally, the L1-L5 vertebrae were individually segmented using region growth. The 3D model was generated, wrapped and subjected to smoothing treatment. The holes were filled and the surface was smoothened and the model was explored and saved in the STL format file.

Geomagic Wrap 2017 modeling process: The STL file was opened using the Geomagic Wrap 2017 software (Raindrop Geomagic Inc., Morrisville, NC, USA) and subjected to surface treatment, which included the removal of features, fast fairing and filling holes. Next, in the precise surface module, contour lines, and surface slices were divided, grilles were generated, NURBS surface entity format was generated, to obtain an STP format.

Solidworks 2023 modeling process: The STP model file saved from Geomagic software was opened using the SolidWorks 2023 software (SolidWorks Corp., Waltham, MA, USA), saved as part format, and assembled. The models of annulus fibrosus, nucleus pulposus, endplate, cartilage, and pedicle nail were constructed using the commands of stretch boss, stretch excise, rotating boss, scanning, isometric surface and segmentation.

Each lumbar vertebra contained cancellous, cortical, and posterior bone components, while the annulus fibrosus encircled the core nucleus of each intervertebral disc. Furthermore, the cartilaginous endplate components were employed to replicate the intervertebral disc and vertebral connection. Anterior longitudinal ligament, posterior longitudinal ligament, ligamentum favum, interspinous ligament, capsular ligaments, and intertransverse ligament were included in the model. The cartilaginous endplate components accurately replicated the intervertebral disc-vertebral connection. The model incorporated all six major lumbar spine ligaments: the anterior longitudinal ligament, posterior longitudinal ligament, ligamentum flavum, interspinous ligament, capsular ligaments, and intertransverse ligament.

The adopted boundary conditions were as follows: movements at the bottom of the sacrum were generated the constraint, and several unified movements were monitored. A compressive preload of 400.0 N combined with a pure moment of 10.0 N.m was applied at the central node on the top side of the first lumbar vertebra to simulate flexion, extension, left–right lateral bending and left–right axial rotation movements.

### Construction and validation

The L1–L3 normal spine model was established, fixing the lower part of the L3 vertebra and establishing a baseline plane. As previously described, we applied a compressive load of 400 N and a torque of 10 N·m to simulate lumbar flexion, extension, lateral bending, and rotational movements. The range of motion was recorded for each direction of movement. The results of this study are similar to those reported in previous literature (9). The similarity of the study results confirms the validity of the model, allowing for further analysis.

### Mesh convergence test

Since this study mainly focused on the safety of internal fixation screws under two types of fracture reduction methods, the maximum equivalent stress of the internal fixation screws under different mesh sizes was compared by adjusting the mesh size of the screws. The mesh sizes were set at 0.5 mm, 1.0 mm, 2.0 mm, 3.0 mm, and 5.0 mm, respectively. The maximum equivalent stress of the screws was selected as the observation index, and the relationship between the stress and the mesh size is shown in Table 1. Based on the calculation efficiency, in the subsequent finite element model research, a mesh size of 1.0 mm was selected as the mesh dimension.

**Table 1:** Material properties of the finite element models.

Component	Young's modulus (MPa)	Poisson ratio
Cortical bone	12,000	0.3
Cancellous bone	100	0.2
Endplate	12,000	0.3
Fibrous rings	6	0.4
Nucleus pulposus	0.0013	0.4999
Cartilage	10	0.3
Titanium alloy	96,000	0.36

### General information

The retrospective study was performed at a single center. All patients who developed single segment thoracolumbar fractures and received the pivot forward combined leveraging technique in Renmin Hospital of Wuhan University during November 2021—January 2025 were enrolled in the study. The study protocol was approved by the Medical Ethics Committee of Renmin Hospital of Wuhan University (WDRY2023-K065) and was performed in line with the relevant guidelines and regulations, and the patient provided an informed consent.

The following inclusion criteria were used: (1) the angle of the injured vertebra was > 20°, and the time from injury to surgery was ≤2 weeks; (2) pivot forward combined leveraging technique and fixation surgery; (3) the major evaluation indicators were imaging parameters before and after surgery, clinical recovery status of thoracolumbar function, among others; (4) retrospective case study with follow-up time exceeding 12 months. The exclusion criteria were: (1) patients who had a history of spinal malformations, infections, tumors, mental abnormalities or neurological diseases; (2) spinal osteoporotic fracture and pathological fracture; (3) those with incomplete and missing imaging and clinical data before and after surgery.

A total of 47 patients with thoracolumbar fracture and segmental angle > 20° underwent the pivot forward combined leveraging technique. Among them were 23 males and 24 females, with ages ranging from 17 to 62 years, with a mean of (46.5 ± 13.4) years. Fracture site: T12 12 cases, L1 16 cases, L2 13 cases, L3 4 cases, L4 2 cases. The causes of injury were as follows: 21 cases of falling, 18 cases of falling from high places, 6 cases of traffic accidents, and 2 cases of heavy objects. The study population demonstrated the following injury distribution according to the AO Spine Thoracolumbar Injury Classification system (10): type A1 (*n* = 13), type A3 (*n* = 26), type B1 (*n* = 4), and type B2 (*n* = 4). The Thoracolumbar Injury Classification and Severity Score (TLICS) (11) averaged 5.7 ± 1.1, with scores spanning from 5 to 8. Neurological function were determined as follows using the ASIA classification: 6 case of grade B, 5 cases of grade C, 8 cases of grade D, and 28 cases of grade E.

### Surgical methods

All patients were placed in prone position and put under general anesthesia. Soft pads were placed on the patients' chest and anterior superior iliac spine, and the abdomen was suspended, to ensure that the thoracolumbar spine remained in an overextended position to promote the injured vertebra reduction.

Utilizing the Wiltse approach, we achieved surgical exposure while preserving neural integrity. The space between the longissimus and the multifidus muscle was bluntly separated, and the outer edge of the transverse process and facet joints was exposed. For patients who required decompression of the laminae, traditional paravertebral dissection was performed.

Universal pedicle screws were implanted into the upper and lower ends of the vertebrae, and uniaxial pedicle screws were implanted into the injured vertebrae. Ensure that the axis of the inserted uniaxial pedicle screws was as parallel as possible to the collapsed endplate. The cap of the screw was higher relative to the cap of the upper and lower pedicle screws. Using the C-arm x-ray machine fluoroscopy, it was observed that the tail caps of 3 pedicle screws exhibited an inverted arc. Notably, the prebending bar angle gradually increased from T11 to L4 as revealed in the examination of the physiological curvature of the thoracolumbar segment. The prebending bar 10°–15° was selected for T11, T12 and L1 segments, and the prebending bar 15°–25° was selected for L2, L3 and L4 segments.

Installation of the precurved connecting rods on both sides. First, the tail cap of the universal pedicle screw was locked on the uninjured endplate vertebra (primarily the distal end). Subsequently, the rod holder was employed as the fulcrum to longitudinally stretch the pedicle screw on the endplate-injured side end vertebra and lock the tail cap. Using the large force-holding bar clamp as the anchor point, the locking sleeve that fixed the injured vertebra serves as the lever force to pry the uniaxial pedicle screws in the direction of the collapsed endplate. The collapsed cartilage endplate was restored and the screw cap is locked in place. The vertebral disc and the anterior midcolumn height of the injured vertebra were elevated longitudinally, and the height of the injured vertebra and the physiological curvature of the spine recovered well (Figure 1). Then was followed by indwelling the negative pressure drain and closure of the incision.

**Figure 1:**
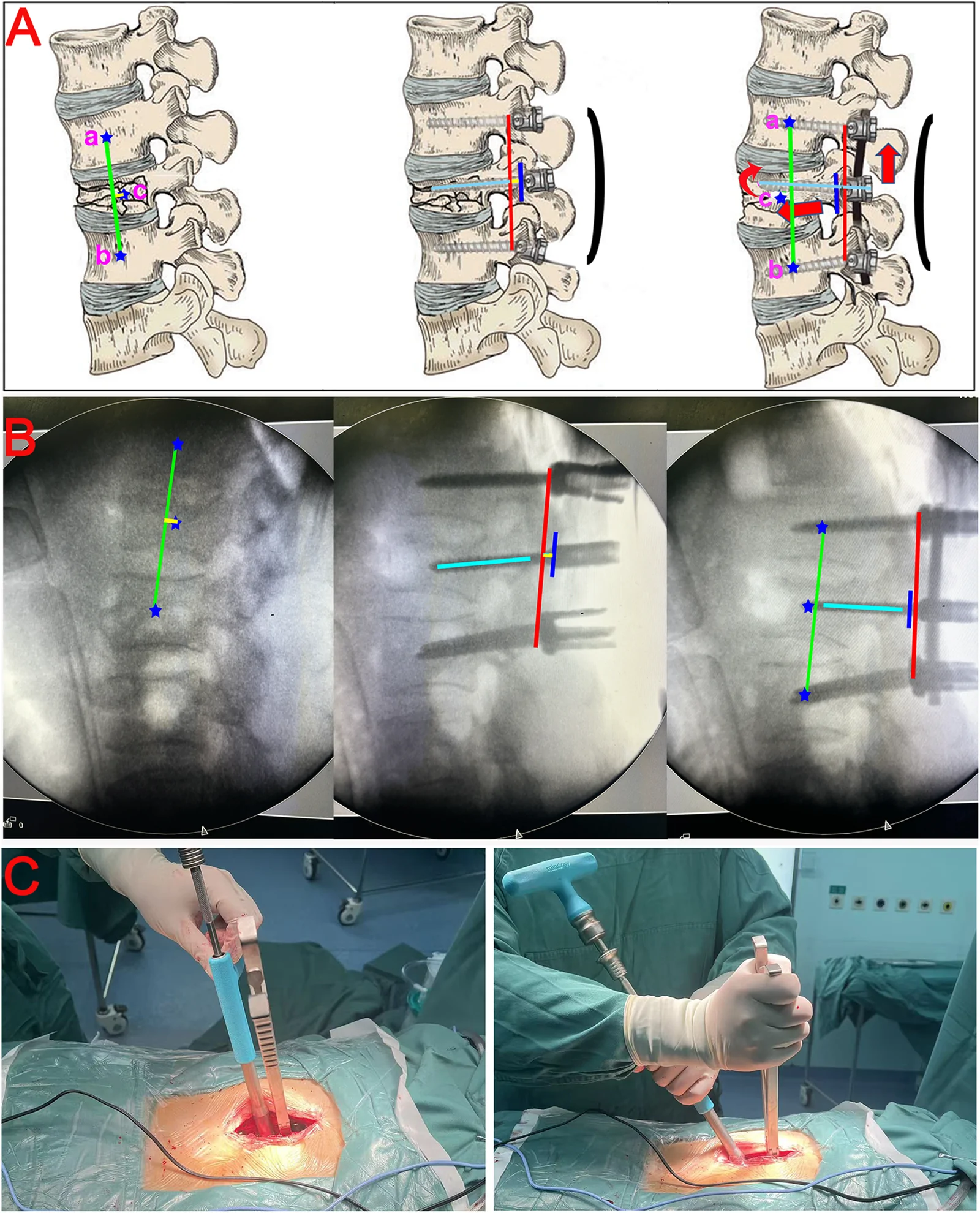
Schematic diagram of the pivot forward combined leveraging technique. **(A)** Blue dots represent center of gravity positions of upper and lower vertebrae (a and b) and injured vertebra (c) Yellow line indicates the distance between center of gravity of injured vertebra and upper and lower vertebrae. The distance between shallow screws of injured vertebra (yellow line) is equal to the distance between center of gravity of injured vertebra and upper and lower vertebra. **(B)** Intraoperative reduction using the pivot forward combined leveraging technique. **(C)** Intraoperative image showing the use of the injured vertebra screw for repositioning.

### Postoperative management

Within the 48–72 h post-surgery, routine prophylactic antibiotics were administered, and drainage tubes were removed if the wound drainage volume was less than 50 ml 48 h after surgery. Anterior-lateral thoracolumbar x-ray films and three-dimensional CT reconstruction were reviewed, which confirmed that the patient's condition was stable and discharged.

### Observation indicators

The key parameters such as, operative time, intraoperative blood loss and pedicle screw placement accuracy were recorded. Radiographs or CT were employed to determine the position of internal fixation and the presence of loosening, displacement, fracture and kyphosis.

### Efficacy evaluation index

The patients underwent regulator follow-up examinations at 1, 3, 6, and 12 months after surgery, and the clinical effect was analyzed based on the Visual Analog Scale (VAS) score (12) and Oswestry Disability Index (ODI) (13). x-ray or CT examinations were performed routinely to obtain the following imaging indicators (Figure 2):

(1) Forward displacement distance of injured vertebral body center: the vertical distance of the line connecting the center of injured vertebral body to the upper and lower vertebral centers, which is equal to the shallow placement distance of pedicle screw of injured vertebral body. The vertical distance from point c at the center of the injured vertebral body to the line connecting a and b (Figure 1);
(2) Vertebral body index (VBI): the ratio of anterior margin height to posterior margin height. A smaller value indicated a greater difference between anterior margin height and posterior margin height of the injured vertebra;
(3) Height of the anterior margin of injured vertebra (HAMIV): the ratio of the height of the injured vertebral front to the reference height of the injured vertebral front. The reference height of the injured vertebral front was defined as the average height of the upper and lower vertebrae near the injured vertebrae. A smaller ratio reflected a larger difference between the height of the injured vertebral front and the ideal height;
(4) Injured vertebra angle (IVA): the angle formed by the extension lines of the upper and lower end plates of the injured vertebra, the larger the value, the greater the angle of the injured vertebra, and the more serious the height loss of the injured vertebra;
(5) Kyphotic Cobb angle (KCA): the angle between the vertical line connecting the upper endplate of the upper vertebra and the lower endplate of the lower vertebra of the injured vertebra.

**Figure 2:**
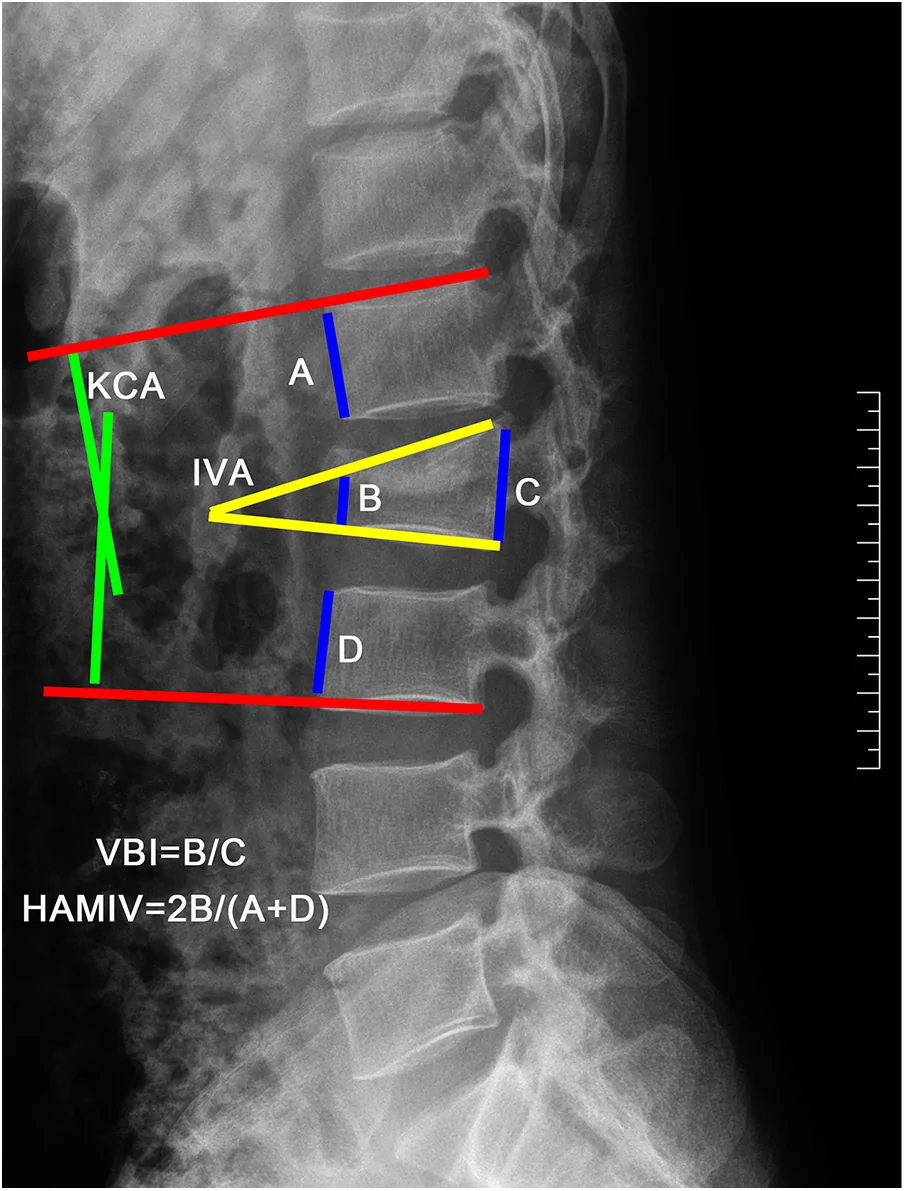
X-ray was performed routinely to obtain the imaging indicators of VBI, HAMIV, IVA, and KCA.

### Statistical analysis

SPSS 25.0 (SPSS, USA) statistical software package was used for statistical analysis, with all two-sided tests set at *α* = 0.05. The measurement data were subjected to the Shapiro–Wilk test for normality, and all of them showed normal distribution. Data were expressed as the mean ± standard deviation (SD). A repeated measures analysis of variance was employed. For comparisons at different time points, the Bonferroni method was used. Effect sizes and 95% confidence intervals (95% CIs) were reported for all core outcomes, rather than *P*-values alone. Continuous outcomes were presented as mean differences (MDs) with 95% CIs. A result was considered statistically significant only if the 95% CI excluded the null value (0 for difference-based measures, 1 for ratio-based measures). Interpretation prioritizes effect magnitude and CI precision.

## Results

### Intact finite element model

To develop the intact L1-L5 finite element model, we utilized Mimics 19.0, Geomagic Wrap 2017, and SolidWorks 2023. The resulting high-quality model included five vertebrae, four intervertebral discs, and six ligaments. The pivot forward combined leveraging technique group contained 566640 tetrahedral elements and 954,749 nodes. In the traditional fixation group, there were 565,919 tetrahedral elements and 955,936 nodes.

### Two finite element models of posterior fixation in L1-L3 vertebrae

The L1-L5 model was selected to replicate the L1-L3 posterior screw rod system fixation procedure based on the validated intact finite element model. Using two finite element models, the simplified pedicle screws and connecting rods were combined into various internal fixation systems once they were designed in Geomagic Wrap 2017. In this case, an infinite friction coefficient was employed to define the contact surfaces between screws and trajectories. Furthermore, each part of the internal fixation system was identified as a whole. The pivot forward combined leveraging technique and typical fixation surgery finite element models with varying pedicle screw locations and orientations were constructed to facilitate biomechanical experiments (Figure 3).

**Figure 3:**
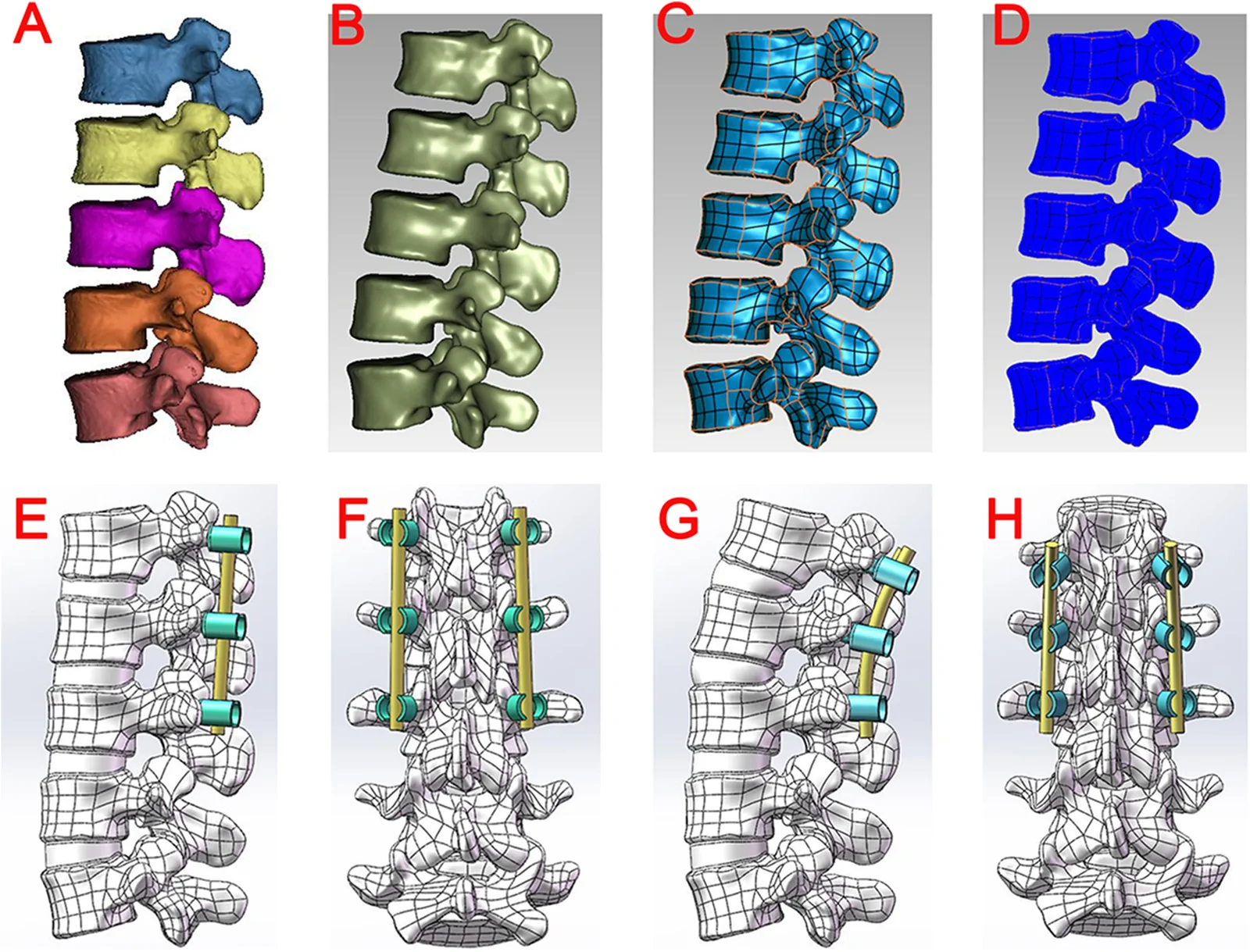
The procedure to establish the intact finite element model of L1-L5 vertebrae. **(A,B)** A rough 3D model of L1–L5 vertebrae. **(C,D)** Model surface treatment and contour line division under the precise surface module. **(E–H)** The model diagrams of the traditional group and the pivot forward technique group created in Solidworks 2023.

The maximal von mises stress of lumbar bone, segmental cartilaginous endplate, annulus fibrosus, nucleus pulposus and facet joints different markedly between the two finite element models. Large stress concentrations were detected on the L3-L4 and L4-L5 intervertebral discs in both postoperative finite element models. Elevated stress concentrated areas were observed on L3-L4 and L4-L5 intervertebral discs in the two postoperative finite element models.

In all models, the maximal von mises stress was concentrated primarily at the centers of the connecting rods and the roots of the pedicle screws, particularly during lateral bending and axial rotating. Interestingly, we observed that the stress distribution in the pivot forward combined leveraging technique tended to be more dispersed compared with the traditional fixation. The mean nail rod stress of the two groups (pivot forward combined leveraging technique and traditional fixation) was 252.18 MPa and 281.85 MPa, respectively. Moreover, the maximum stress level for pivot forward combined leveraging technique and traditional fixation systems was approximately 303.74 MPa and 355.28 MPa, respectively.

The stress on the nucleus pulposus in the pivot forward combined leveraging technique was lower than that observed in traditional fixation systems. Furthermore, the average nucleus pulposus stress in pivot forward combined leveraging technique and traditional fixation systems was 0.00097 MPa and 0.00111 MPa, respectively. The value of nail rod stress and nucleus pulposus stress were shown in Figures 4, 5.

**Figure 4:**
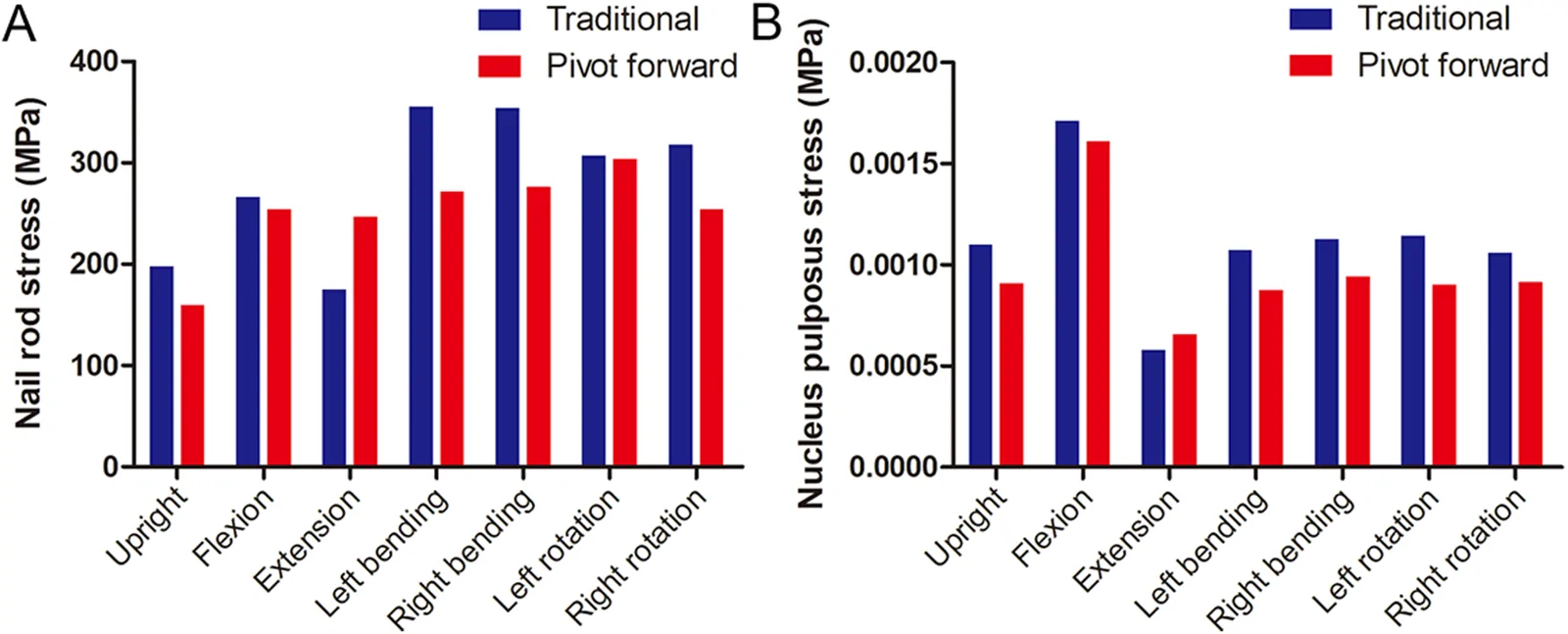
Comparison of the nail rod stress **(A)** and nucleus pulposus stress **(B)** of pivot forward technique and traditional fixation systems.

**Figure 5:**
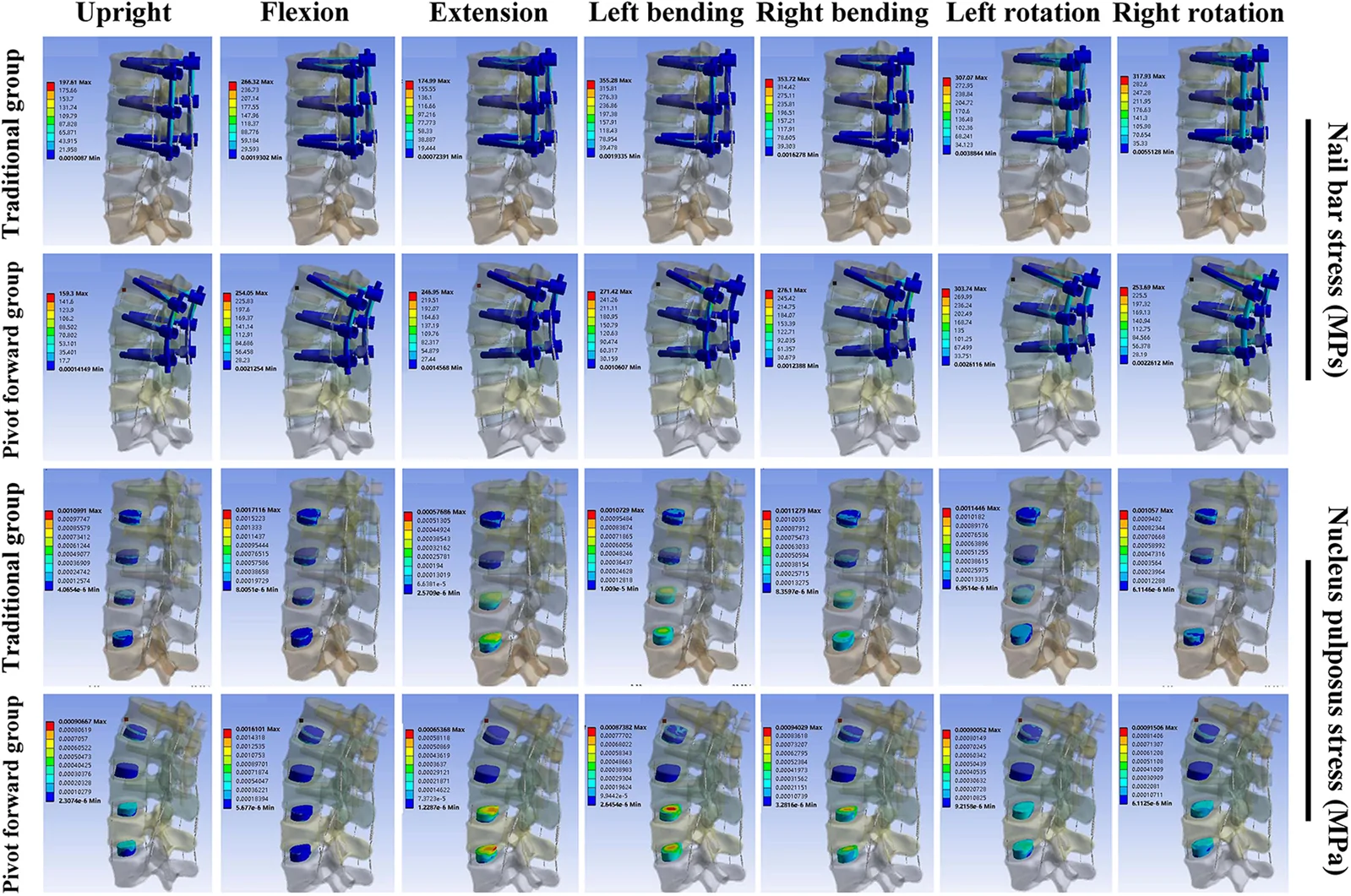
The nail rod stress and nucleus pulposus stress distribution of screw-rod systems in two postoperative finite element models under different conditions. According to the indicator diagram, red indicates the stress concentration area, while blue shows the stress dispersion area.

### Demographic and operative data

The enrolled 47 patients were successfully operated and 282 pedicle screws were successfully inserted, through an operation time was (85.00 ± 13.15) min. The intraoperative blood loss was (201.70 ± 48.38) ml. No injuries to the spinal cord, nerves, or major blood vessels occurred during pedicle screw placement. All patients' wounds healed at stage I.

During the superficial pedicle screw compression reduction process of injured vertebrae, the forward displacement distance of the center of injured vertebrae was 2.62 mm∼ 3.51 mm, with an average of (3.00 ± 0.23) mm. The thread spacing of the pedicle screw was approximately 1 mm, and the shallow placement distance of the injured pedicle screw was about 2 to 3 thread spacings.

### VBI

Prior to operation, the VBI of 47 patients was 0.64 ± 0.07, which increased significantly to 0.93 ± 0.04 post-surgery, representing an improvement rate of the postoperative VBI was 80.6%. The VBI decreased slightly at the last follow-up (0.91 ± 0.04), but not significantly compared to the level of post-operation (*P* = 0.126, Table 2). The improvement rate of VBI at the last follow-up was 75.0%.

**Table 2:** Comparison of imaging parameters and clinical outcomes before and after surgery in 47 patients $\left(\bar{\chi }\pm s\right)$.

Evaluation index	Pre-op.	Post-op.	Last follow-up	Pre-op. vs. Post-op.	95% CI [lower limit, upper limit]	Pre-op. vs. Last follow-up	95% CI [lower limit, upper limit]	Post-op. vs. Last follow-up	95% CI [lower limit, upper limit]
VBI	0.64 ± 0.07	0.93 ± 0.04	0.91 ± 0.04	0.000	−31.571, −26.573	0.000	−29.454, −24.457	0.126	−0.382, 4.616
HAMIV	0.60 ± 0.09	0.96 ± 0.04	0.94 ± 0.04	0.000	−38.216, −31.984	0.000	−36.170, −29.937	0.341	−1.070, 5.163
IVA (°)	23.70 ± 3.63	4.22 ± 2.32	5.49 ± 2.32	0.000	16.881, 19.703	0.000	15.615, 18.437	0.094	−19.703, −16.881
KCA (°)	11.37 ± 9.53	−1.71 ± 8.07	0.18 ± 8.25	0.000	8.766, 17.407	0.000	6.876, 15.517	0.873	−6.211, 2.431
VAS score	8.04 ± 0.83	3.26 ± 0.64	1.00 ± 0.78	0.000	4.409, 5.165	0.000	6.665, 7.420	0.000	1.877, 2.633
ODI (%)	51.81 ± 9.21	21.15 ± 5.52	6.49 ± 1.90	0.000	27.514, 33.806	0.000	42.173, 48.465	0.000	11.514, 17.806

### HAMIV

The HAMFV in 47 patients was 0.60 ± 0.09 prior to surgery, which was significantly increased to 0.96 ± 0.04 post-surgery (*P* = 0.000, Table 2). This represented an improvement rate of 90% for HAMFV. At the last follow-up, the HAMFV decreased slightly, to 0.94 ± 0.04, and the height was lost by about 3.1%, but the difference was no statistically significant compared to level of post-operation (*P* = 0.341, Table 2). The improvement rate of the HAMFV at the last follow-up was 85.0%.

### IVA

In 47 patients, the IVA was 23.70° ± 3.63° before surgery, which significantly decreased to 4.22° ± 2.32° post-surgery (*P* = 0.000, Table 2), translating to an improvement rate of IVA of 82.2%. At the last follow-up, the IVA was slightly lost (5.49° ± 2.32°), but not significantly compared to the post-operative results (*P* = 0.094, Table 2). The last follow-up identified an angle improvement rate of 76.8% for IVA.

### KCA

The KCA of 47 patients was 11.37° ± 9.53° prior to operation, which significantly decreased to −1.71° ± 8.07° post-surgery (*P* = 0.000, Table 2). The improvement rate of KCA after surgery was 115.0%. At the last follow-up, the KCA increased slightly, to 0.18° ± 8.25°, but it was not significantly different from post-operative results (*P* = 0.873, Table 2). The improvement rate of KCA at the last follow-up was 98.4%.

### Postoperative VAS score and ODI value

The analysis revealed that the VAS score of 47 patients decreased from the preoperative period (8.04 ± 0.83) to the post-operative period (3.26 ± 0.64). Notably, the postoperative VAS score was significantly different relative to levels before surgery (*P* = 0.000, Table 2), with low back pain showing an improvement rate of 59.5%. At the last follow-up, the improvement was 1.00 ± 0.78 points, and the difference between the score at the last follow-up and that post-operation was statistically significant (*P* = 0.000, Table 2). It was also observed that the back pain at the last follow-up was 87.6%, suggesting that the symptoms of low back pain continued to improve during the follow-up period.

The ODI of 47 patients increased from (51.81 ± 9.21) % before surgery to (21.15 ± 5.52) % post-surgery, and was improved to (6.49 ± 1.90) % at the last follow-up. Statistically significant differences were observed between preoperative and postoperative measurements, between preoperative and last follow-up measurements, and between postoperative and last follow-up measurements (*P* = 0.000, Table 2). The improvement rate of thoracolumbar function after surgery was 59.2% and 87.5% at post-surgery and at the last follow-up, suggesting that thoracolumbar function exhibited a marked improvement during the follow-up. Representative case who underwent an operation via pivot forward combined leveraging technique is presented in Figure 6.

**Figure 6:**
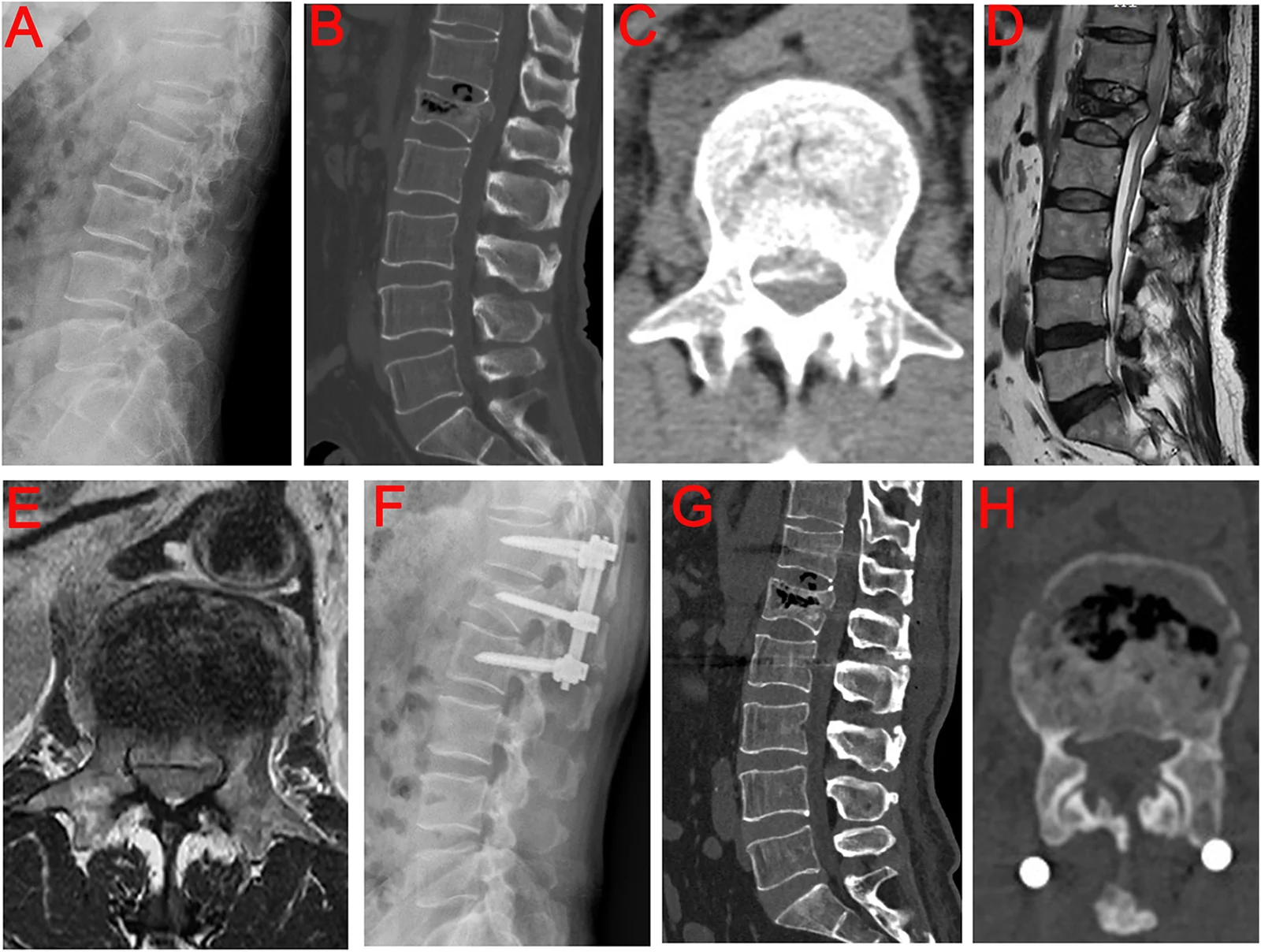
Pre-, intra-, and post-operative radiographs treated with pivot forward combined leveraging technique. **(A)** Preoperative x-ray. **(B,C)** Preoperative CT sagittal and transverse plane view. **(D,E)** Preoperative MRI sagittal and transverse plane view. **(F)** Postoperative lateral x-Ray. **(G,H)** Postoperative CT sagittal and transverse plane view.

## Discussion

Surgical treatment for thoracolumbar spinal fractures aims to restore spinal stability and alignment, as well protect neurological function (14). To facilitate early ambulation and function recovery, it is necessary that the selected surgical approach should treat segmental kyphotic deformity and avoid late instability and pain. Although short segment fixation can effectively treat thoracolumbar injuries, it can potentially increase the risk of hardware failures and kyphosis recurrence, either with or without clinical consequences (15). This increases the possibility of a biomechanical failure as a function of inadequate anterior support and less reliable instrumentation (16). Modifications in short segment fixation can reduce failure rates. However, short segment instrumentation is still the preferred approach by surgeons in managing most thoracolumbar injuries owing to its ease of operation acceptable clinical results (17, 18).

It has been reported that the combination of postural reduction, pre-contouring of rods, and cantilever correction can reduce fractures in short segment fixation (19). Given the damaged endplate, careful distraction is advocated. The screw-bone contact may experience stress while kyphotic angulation may be aggravated by excessive distraction, causing a differential increase in distraction over the posterior column (20, 21). The application of screws in fractured vertebrae is commonly adopted to optimize the efficacy of short segment instrumentation (22).

The biomechanical principle of pivot forward combined leveraging technique can be defined in the following four aspects: (1) the pedicle screw of the injured vertebra serves as the lever fulcrum, and the whole injured vertebra is pressed and pushed on the sagittal plane, so that the front and middle columns are fanned out, and the center of gravity of the injured vertebra is shifted forward; (2) moderate lordotic prebending with the connecting rod can further enhance the pedicle screw fulcrum effect of the injured vertebra and restore the physiological curvature of the spine; (3) the end of the vertebral uniaxial screw in entire injured vertebra parallel propulsion, longitudinal lifting of the intervertebral disc and compression, collapse of the vertebral body front and middle column height; (4) the collapsed vertebrae endplates were restored to a good level of flatness through the direct distraction of the uniaxial pedicle screws. It is particularly indicated for thoracolumbar fractures presenting with severe vertebral height loss. When applying the pivot forward combined leveraging technique, two strong forces of fan-shaped and paralleloidal were formed via mechanical analysis. The shallow placement amplitude of the injured vertebra and the pre-bending curvature of the connecting rod were determined based on the degree of collapse of the injured vertebra, the angle of segmental kyphosis and the original physiological curvature of the injured vertebra. During distraction of the connecting rod and compression of the injured vertebra, the fan-shaped and parallel distraction forces simultaneously facilitated vertebral reduction, preservation of physiological spinal curvature, restoration of vertebral height, and recovery of the flatness of the endplate of the fractured vertebra. After compressing the injured vertebra, for the central depressed area of the endplate, a uniaxial pedicle screw was used to pry the collapsed area of the endplate, thereby restoring the flatness of the endplate surface. The technique of compressive distraction and repositioning of the injured vertebrae is complementary to each other in restoring the height of the injured vertebrae and the smoothness of the collapsed endplates, achieving excellent repositioning and fixation results. This combined biomechanical effect enabled effective correction of kyphosis deformity and the reconstruction of sagittal balance.

During the reduction of the injured vertebra's fulcrum forward, the lack of top pressure can limits the reduction of the vertebral height and improvement of physiological curvature following the injured vertebra's center of gravity. However, excessive top pressure cause pronounced anterior movement of injured vertebrae, overcorrection, and even secondary nerve root canal stenosis and nerve irritation. To prevent the development of excessive or insufficient compression and distraction, we adjusted the degree of shallow placement of the injured vertebra to meet the reduction needs of different degrees of vertebral fractures with reference the position of the center of gravity and physiological curvature of the injured vertebra. The depth of implantation of the injured vertebra screw was measured and the vertical distance between the center of gravity of the injured vertebra and the center of gravity of the lower and upper vertebra before surgery was designated as the shallow height of the pivot screw of the injured vertebra. By applying pressure to the injured vertebra, its center of gravity was shifted forward toward the center of gravity of the adjacent upper and lower vertebra. Subsequently, the rod-bending technique was employed to further advance the center of gravity of the injured vertebra, aiding in the restoration of the spinal natural curvature. In this study, the distance of the superficial placement of the pedicle screw of the injured vertebra was accurately measured using the preoperative x-ray images. The working distance for the top compression reduction of the injured vertebra was exactly equal to the superficial placement distance of the pedicle screw. The anterior displacement distance of the injured vertebral body center was in the range of 2.62 mm to 3.51 mm, with an average value of (3.00 ± 0.23) mm. The pitch of the pedicle screw threads was approximately 1 mm, suggesting that the shallow placement distance of the injured pedicle screw was nearly 2 to 3 screw pitches. The prebending angle of the connecting rod can be adjusted with reference to the center of gravity of the injured vertebra and the initial physiological curvature of the spine, to achieve good reduction and fixation.

Reserch has shown that the sagittal Cobb angle can serve as an index for evaluating the spine sagittal balance (23). In this study, the forward pivot distance of the injured vertebra was quantified during the reduction of a single thoracolumbar fracture. Of note, the vertical distance between the center of gravity of the injured vertebra and the center of gravity of the upper and lower vertebra reflects the working distance of the top pressure of the injured vertebra and the distance of the shallow placement of the injured vertebra. With reference to the normal physiological curvature of the thoracolumbar segment, we precurved the joint rod and utilized the curved joint rod to maintain the physiological curvature of the injured segment.

Furthermore, this study found that at the last follow-up, the VBI, HAMIV, IVA, and KCA indicators had decreased compared to those immediately after the surgery, although there was no statistically significant difference. This indicates that there was a loss of vertebral height in the injured vertebrae or a mild local kyphosis phenomenon in the later stage. This is a common phenomenon in clinical practice. The reasons for the analysis mainly include: (1) apoptosis or necrosis of nucleus pulposus and annulus fibrosus cells, resulting in narrowing of the intervertebral space; (2) “empty shell phenomenon” within the vertebrae after reduction, with loss of vertebra height; (3) osteoporotic changes in the vertebrae.

At the same time, we noticed that when collecting clinical case data according to the inclusion and exclusion criteria, there was heterogeneity in the study population, mainly due to different types of AO fractures and different neurological conditions. The reason why this study combined these patients into a single cohort was mainly to consider that the majority of patients had fractures of type A1 and A3 according to the AO classification, accounting for 83% of the total. Additionally, the ASIA neurological function evaluation was mainly at levels D and E, accounting for 76.6% of the total. The AO fracture classification and neurological function evaluation mostly focused on the common fracture types and neurological status manifestations in clinical practice. A small number of patients had severe fracture classification and neurological function at level B. We paid more attention to the evaluation of fracture reduction quality after surgery, including indicators such as VBI, HAMIV, IVA, and KCA. These indicators highlight the advantages of the research technique in fracture reduction and reduce the impact of this heterogeneity on the research results. The insertion of the injury vertebra screws was currently a common method for three-column stability and did not interfere with the reduction of the fracture. The direction of the injury vertebra screw insertion was parallel to the collapsed endplate, which was conducive to the maximum levering, especially suitable for the fracture type with collapsed endplate, and particularly suitable for the reduction of OA Type A fractures. In future research, we will collect more patients, fully consider the heterogeneity of the research population, and make the research conclusion more objective and scientific.

Nevertheless, this study has several limitations that need to be clarified. This study included small number of patients, all with single-level fractures; therefore, the effectiveness of the pivot forward combined leveraging technique for treating more complex thoracolumbar fractures remains unclear. Moreover, we did not consider other variables that may potentially influence the therapeutic effect of this reduction technique, such as the cause of the fracture and other types of fracture. Therefore, the level of evidence in this retrospective study is relatively low, and a prospective, multicenter randomized clinical trial with a larger sample size and longer follow-up is recommended to obtain more accurate results.

## Conclusion

Our study indicates that the use of pivot forward combined leveraging technique in the treatment of thoracolumbar fracture can restore the height of the injured vertebra and correct the kyphosis angle of the injured vertebra, achieving good clinical effect.

## Patient's consent

This study was conducted in accordance with the ethical standards of the institutional and/or national research committee and with the 1,964 Helsinki declaration and its later amendments or comparable ethical standards. Written informed consent was obtained from all individual participants included in the study.

## Data Availability

The raw data supporting the conclusions of this article will be made available by the authors, without undue reservation.
